# Comparison of Estimates between Cohort and Case–Control Studies in Meta-Analyses of Therapeutic Interventions: A Meta-Epidemiological Study

**DOI:** 10.1371/journal.pone.0154877

**Published:** 2016-05-09

**Authors:** Amy Lanza, Philippe Ravaud, Carolina Riveros, Agnes Dechartres

**Affiliations:** 1 Mailman School of Public Health, Columbia University, New York, United States of America; 2 INSERM U1153, Paris, France; 3 Université Paris Descartes - Sorbonne Paris Cité, Paris, France; 4 Assistance Publique-Hôpitaux de Paris, Hôpital Hôtel-Dieu, Centre d'Epidémiologie Clinique, Paris, France; 5 Cochrane France, Paris, France; University of New South Wales, AUSTRALIA

## Abstract

**Background:**

Observational studies are increasingly being used for assessing therapeutic interventions. Case–control studies are generally considered to have greater risk of bias than cohort studies, but we lack evidence of differences in effect estimates between the 2 study types. We aimed to compare estimates between cohort and case–control studies in meta-analyses of observational studies of therapeutic interventions by using a meta-epidemiological study.

**Methods:**

We used a random sample of meta-analyses of therapeutic interventions published in 2013 that included both cohort and case–control studies assessing a binary outcome. For each meta-analysis, the ratio of estimates (RE) was calculated by comparing the estimate in case–control studies to that in cohort studies. Then, we used random-effects meta-analysis to estimate a combined RE across meta-analyses. An RE < 1 indicated that case–control studies yielded larger estimates than cohort studies.

**Results:**

The final analysis included 23 meta-analyses: 138 cohort and 133 case–control studies. Treatment effect estimates did not significantly differ between case–control and cohort studies (combined RE 0.97 [95% CI 0.86–1.09]). Heterogeneity was low, with between–meta-analysis variance τ^2^ = 0.0049. Estimates did not differ between case–control and prospective or retrospective cohort studies (RE = 1.05 [95% CI 0.96–1.15] and RE = 0.99 [95% CI, 0.83–1.19], respectively). Sensitivity analysis of studies reporting adjusted estimates also revealed no significant difference (RE = 1.03 [95% CI 0.91–1.16]). Heterogeneity was also low for these analyses.

**Conclusion:**

We found no significant difference in treatment effect estimates between case–control and cohort studies assessing therapeutic interventions.

## Introduction

Randomized controlled trials (RCTs) are traditionally considered the standard for assessing the effects of a healthcare intervention. The recent interest in comparative effectiveness research has emphasized the use of observational studies in assessing treatment effectiveness [1,2]. Observational studies may be more applicable in real-world settings than RCTs because of their broader range of participants included, larger sample size and longer follow-up [1,3,4]. Also, such studies can complement gaps when RCTs are not feasible, and they have lower costs [1,3,4].

Multiple studies have been conducted to compare the results of RCTs and observational studies [2,4–6], summarized in a recent systematic review of the literature [7]: analysis of 1,583 meta-analyses revealed little evidence for significant effect-estimate differences between RCTs and observational studies [7].

By contrast, despite the diversity of observational studies, few studies have investigated differences in treatment effects among observational study types [8]. One meta-epidemiological study showed slightly larger estimates for adverse effects, although not significant, with case–control than cohort studies [8]. Case–control studies are generally considered to have higher risk of bias than cohort studies. They are susceptible to selection bias and to recall bias because cases and controls may not have equal opportunities for the ascertainment of exposure.

In this study, we performed a meta-epidemiological study to compare treatment effect estimates between cohort and case–control studies in a collection of meta-analyses of binary outcomes for therapeutic interventions.

## Material and Methods

### Study design

This is a meta-epidemiological study using a sample of meta-analyses of observational studies assessing binary outcomes for therapeutic interventions.

### Data sources and searches

On January 7, 2014, we conducted a search on MEDLINE via PubMed using a search equation combining keywords and Mesh terms to identify reports of systematic reviews with meta-analysis that included observational studies published in 2013 (Search strategy is reported in S1 Table).

### Study Selection

#### Relevant systematic reviews and meta-analyses

In a first step, one reviewer (CR) reviewed titles and abstracts and the full text when necessary to select all systematic reviews with meta-analysis published in 2013 that assessed, a therapeutic or preventive intervention (ie, vaccine) for efficacy or safety and including observational studies. In a second step, a second reviewer confirmed the eligibility of the pre-selected sample and identified all systematic reviews with meta-analysis of binary outcomes that included data from at least 3 studies, with at least 1 cohort study and at least 1 case–control study. We also included meta-analyses with case-control and cohort studies analyzed separately. Meta-analyses with no comparison group were excluded. Because of the number of systematic reviews and meta-analyses retrieved, we randomly selected a sample of 25 meta-analyses for analysis. This choice was not based on a formal sample size calculation but rather to have a convenient sample.

#### Selection of outcomes

If the systematic review reported meta-analysis results for more than 1 binary outcome, we selected the outcome with the largest number of patients.

#### Individual observational studies analyzed

All individual cohort and case–control studies included in the selected meta-analyses were selected. We selected both prospective and retrospective cohorts and excluded cross-sectional studies. Studies without any events in both groups did not contribute to the analysis.

### Data extraction

Two data collection forms were used: one to collect information about the meta-analysis and one about the cohort or case–control studies included in meta-analyses.

#### Meta-analyses

From each meta-analysis selected, we collected data on the date of publication, funding source, sample size, and number and type of included observational studies. We also recorded the condition analyzed, the intervention assessed for both experimental and control groups, whether the intervention was pharmacologic or nonpharmacologic, and the outcome evaluated. Finally, we extracted the combined meta-analysis results for both crude and adjusted estimates, if available.

#### Observational studies

Whenever possible, we retrieved the original article for each included observational study within the meta-analyses selected and extracted data from both this report and the systematic review report. For each observational study, we collected data on the date of publication, funding source, sample size, and type of observational study (cohort or case–control). For cohort studies, we also collected whether the study was prospective or retrospective. To do so, we relied on the classification of non-randomized studies provided by Ioannidis and colleagues [9]. We defined a prospective cohort study as one in which all subjects are recruited and evaluated prospectively and a retrospective cohort study as one in which subjects are evaluated retrospectively [9].

Both the crude and adjusted results for each observational study for the outcome of interest were recorded, if available. If the adjusted OR was available, we recorded the adjustment variables as well.

### Data synthesis and analysis

#### Data synthesis

We first repeated all meta-analyses using the data reported by the authors and a random effects model. This choice may result in discrepancies with the results reported in the original meta-analysis report if a fixed-effect model was used. We used the measure of the estimate reported in the meta-analysis (ie, relative risk or odds ratio [OR]). We analysed adjusted estimates when available. If the adjusted estimate was not available, the crude estimate was used. Heterogeneity across studies was assessed with the I^2^ statistic. We defined substantial heterogeneity as I^2^≥50%. I^2^ is the percentage of the variability in effect estimates that is due to heterogeneity rather than sampling error (chance).

#### Meta-epidemiological analysis

Our hypothesis was that case-control studies may show larger benefits but also larger harms than cohort studies. Meta-analyses including observational studies generally aim to assess the efficacy of interventions when randomization is difficult to perform (eg, for some surgical interventions) or to assess harms. In the latter situation, the objective of the studies is to reveal harms related to the intervention so our assumption was that the bias would be in the direction of showing more harms. The meta-epidemiological analysis involved the two-step method described by Sterne et al. [10] to estimate differences in treatment effect estimates between case–control and cohort studies. In a first step, for each individual meta-analysis, we estimated the ratio of estimates (RE)–the ratio between the estimate in case–control studies to that in cohort studies. The RE was estimated with random-effects meta-regression analysis to incorporate between-study heterogeneity. In a second step, we estimated a combined RE across meta-analyses and the 95% CI using meta-analysis methods with inverse variance weighting and random-effects between meta-analyses using the moment-based variance estimator. Because we hypothesized that case–control studies would show larger estimates of benefit or adverse events than cohort studies, we re-coded outcomes so that a RE < 1 indicated that case–control studies yielded larger estimates of the intervention effect or adverse events than cohort studies. Heterogeneity across REs was assessed by the between–meta-analysis variance τ^2^.

#### Subgroup and sensitivity analyses

In a sensitivity analysis, we used only the adjusted estimates available for the cohort and case–control studies. Additionally, in 2 secondary analyses, we stratified by prospective or retrospective cohort studies to ensure the robustness of our results.

All analyses involved use of STATA SE 11.0 (StataCorp, College Station, TX), with metan and metareg subroutines.

## Results

### Characteristics of included meta-analyses

**Fig 1:**
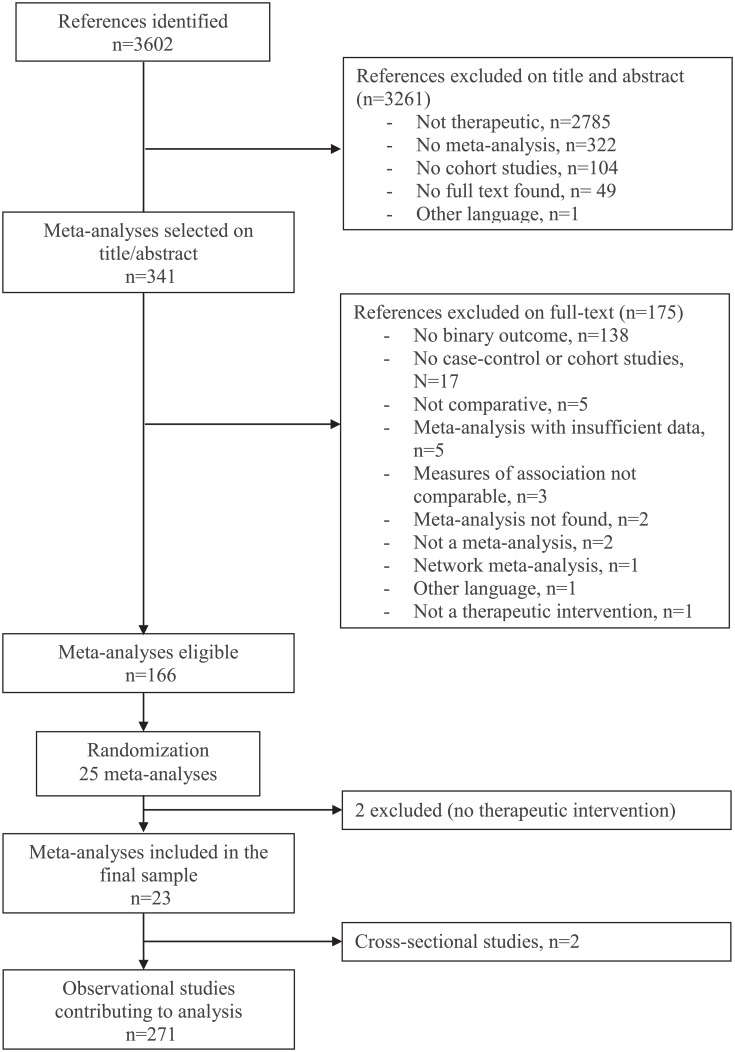
Flow chart of the selection of meta-analyses.

Of the 3,602 meta-analyses identified by the electronic search, 166 were eligible for analysis (Fig 1). We initially obtained a random sample of 25 meta-analyses but 2 were ineligible, so 23 remained in the final sample [11–33]. S2 Table gives the characteristics of included meta-analyses.

The number of included observational studies was 271; the median number of observational studies per meta-analysis was 9 (range 3–32). The combined treatment effect estimates from individual random-effects meta-analyses ranged from 0.28 to 3.97. Overall, 17 of 23 meta-analyses showed substantial heterogeneity (*I*^*2*^ ≥ 50%).

A total of 21 meta-analyses concerned pharmacological interventions [10–17,19–30,32,33], for 255 observational studies (median number of studies in these meta-analyses, 9 [range 6–32]). Two meta-analyses concerned nonpharmacological interventions [18,31], for 16 observational studies (median number of studies, 8 [range, 3–13]).

### Characteristics of observational studies

Among the 271 observational studies included, 133 (49%) were case–control studies and 138 (51%) cohort studies (Table 1). The case–control and cohort studies differed largely in sample size, with a median sample size of 767 (Q1-Q3: 206–2332) and 4700 (Q1-Q3: 501–51000), respectively. Results were more frequently reported as ORs for case–control than cohort studies (72% vs 49%). Nevertheless, in all meta-analyses except 1, the treatment effect measure reported by the review authors was the same for all studies within the same meta-analysis. Reporting of adjusted estimates was more common for case–control than for cohort studies (79% vs 66%). Private funding was less common for case–control than cohort studies (27% vs 42%).

**Table 1:** Characteristics of cohort and case–control studies.

Characteristics	Case–control studies	Cohort studies
	n (%)	n (%)
	n = 133	n = 138
**Type of intervention**		
Pharmacological	124 (93)	131 (94)
Nonpharmacological	9 (7)	7 (6)
**Funding source**		
Public	24 (19)	15 (11)
Private	34 (27)	56 (42)
Both public and private	19 (15)	17 (13)
Unclear	7 (6)	10 (8)
Not reported	42 (33)	35 (26)
**Setting**		
Single-center	38 (29)	35 (25)
Multicenter	88 (66)	99 (72)
Not reported	7 (5)	4 (3)
**Sample size**		
Median (Q1-Q3)	767 (206–2332)	4700 (501–51000)
**Measure used**		
Odds ratio	96 (72)	68 (49)
Relative risk	32 (24)	68 (49)
Unclear	5 (4)	2 (2)
**Type of estimates used**		
Adjusted	105 (79)	91 (66)
Crude	28 (21)	47 (34)

### Differences in treatment effect estimates between case–control and cohort studies

**Fig 2:**
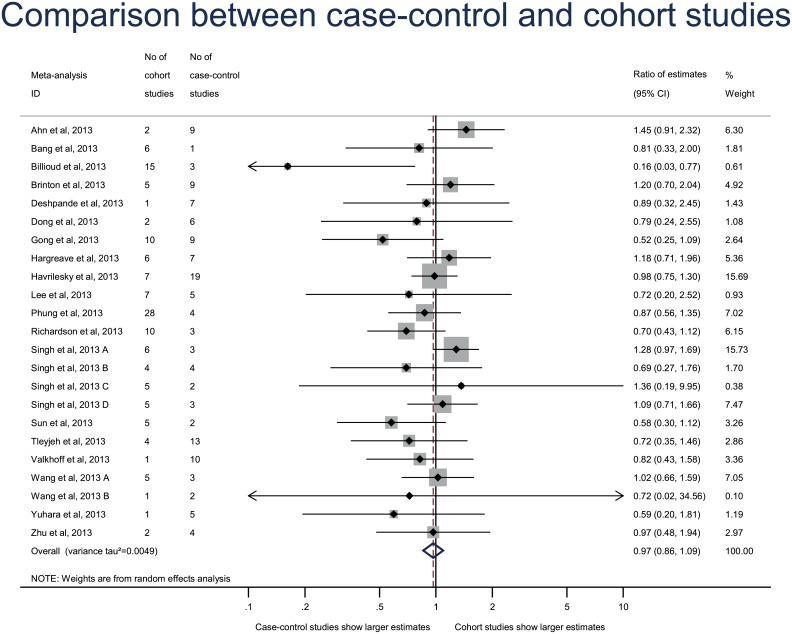
Difference in treatment effect estimates between 133 case–control and 138 cohort studies. Difference in treatment effect estimates is expressed as ratio of estimates (RE). A RE < 1 indicates that case–control studies yielded larger estimates of the intervention effect or adverse events than cohort studies.

In the primary analysis, treatment effect estimates did not differ between case–control and cohort studies (combined RE, 0.97 [95% CI 0.86–1.09], p = 0.58) (Fig 2). The REs were > 1 in 7 meta-analyses and < 1 in 16 and ranged from 0.16 to 1.45. Heterogeneity was low across individual meta-analyses (between meta-analysis variance τ^2^ = 0.0049).

#### Sensitivity and secondary analyses

**Fig 3:**
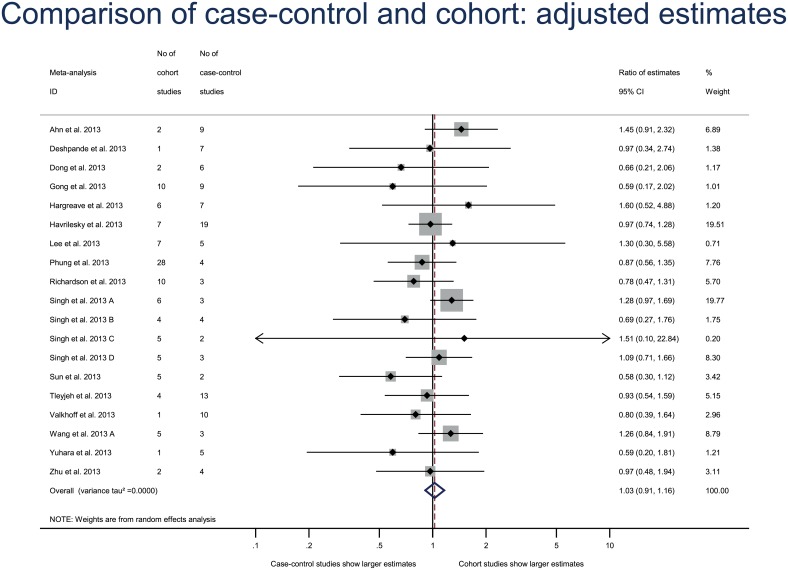
Sensitivity analysis of differences in effect estimates between case–control and cohort studies with available data on adjusted estimates. Difference in treatment effect estimates is expressed as ratio of estimates (RE). A RE < 1 indicates that case–control studies yielded larger estimates of the intervention effect or adverse events than cohort studies.

**Fig 4:**
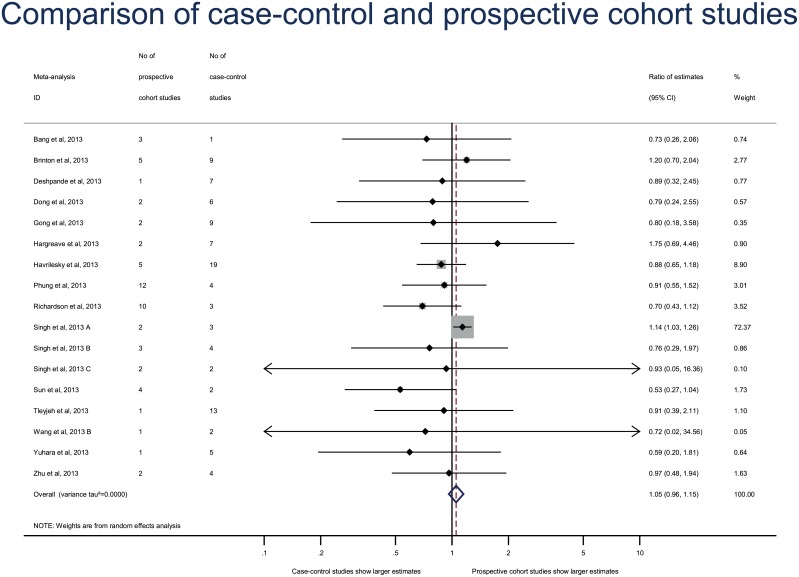
Secondary analysis of differences in effect estimates between case–control and prospective cohort studies. Difference in treatment effect estimates is expressed as ratio of estimates (RE). A RE < 1 indicates that case–control studies yielded larger estimates of the intervention effect or adverse events than prospective cohort studies.

**Fig 5:**
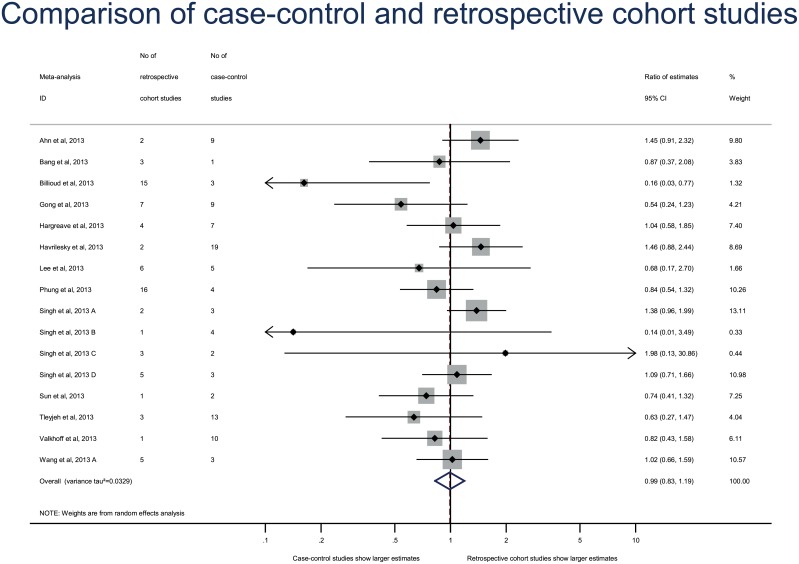
Secondary analysis of differences in effect estimates between case–control and retrospective cohort studies. Difference in treatment effect estimates is expressed as ratio of estimates (RE). A RE < 1 indicates that case–control studies yielded larger estimates of the intervention effect or adverse events than retrospective cohort studies.

Repeating the primary analysis with the adjusted estimates only for both case–control and cohort studies yielded results that were consistent with the original analysis (combined RE, 1.03 [95% CI 0.91–1.16], between–meta-analysis variance τ^2^ = 0.0000) (Fig 3). Another sensitivity analysis excluding the meta-analysis with both relative risks and ORs reported gave consistent results (combined RE 0.95 [95% CI 0.85–1.07], between-meta-analysis variance τ^2^ = 0.000). The estimates did not differ between case–control and cohort studies with both the prospective cohort analysis (combined RE, 1.05 [95% CI 0.96–1.15], between–meta-analysis variance τ^2^ = 0.000)(Fig 4) and retrospective cohort analysis (combined RE, 0.99 [95% CI 0.83–1.19], between–meta-analysis variance τ^2^ = 0.0329)(Fig 5).

## Discussion

This meta-epidemiology study compared treatment effect estimates between cohort and case–control studies with binary outcomes in a sample of meta-analyses covering a wide range of therapeutic interventions. Overall, we found no statistically significant differences between cohort and case–control studies. Similarly, sensitivity analysis with adjusted estimates only and secondary analyses with prospective and retrospective cohort studies revealed no significant differences in effect esimates.

The present study adds to and extends existing literature comparing treatment effect estimates by study design. Because of the prominence of the RCT design in healthcare interventions, the drawbacks and advantages of this design have been covered extensively in the methodological literature. The RCT, as well as meta-analyses of studies of this design, provides the most reliable estimates [34]. One systematic review of differences in treatment effect estimates between observational studies and RCTs suggested little evidence for significant effect-estimate differences [7]. Observational studies are increasingly being used for assessing therapeutic interventions when RCTs are difficult or impossible to conduct or when assessing safety. Only one study compared treatment effect estimates between cohort and case–control studies in evaluating adverse effects and found slightly higher estimates of harm in case–control than other observational studies [8].

The limitations of our study should be considered. We performed a search for all meta-analyses including observational studies for therapeutic interventions available in 2013 in PubMed. Our search is not entirely exhaustive, but we needed only a relatively representative sample of meta-analyses available to the clinicians. To have a convenient sample, we randomly selected 25 meta-analyses, so our sample size is rather small which may limit the power of our analysis and the generalisability of our findings. We did not perform a formal sample size calculation because this is complex for meta-epidemiological studies and because of the uncertainty regarding the proportion of cohort and case-control studies within meta-analyses and the amount of difference in treatment effect estimates [35]. Although we also considered meta-analyses analyzing separately cohort and case-control studies, only one was included in our sample. So, we cannot exclude that for the other meta-analyses, the review authors considered it appropriate to combine results of cohort and case-control studies because their results were not too different. The model we used assumes a similar level of heterogeneity for case-control and cohort studies. A Bayesian hierarchical model may allow for modeling the average increase in between-study heterogeneity in studies with a specified study design, namely, case-control studies [36]. We did not use this model because we have no evidence to support that the variance between studies would be higher for case-control than for cohort studies. Finally, meta-confounding must be considered. Although it is hard to control for, we attempt to account for this with sensitivity and secondary analyses.

Our study has many implications for insights into the methodological literature on study design. The Cochrane Collaboration has developed a tool for assessing risk of bias in RCTs, the Risk of Bias tool [34]. This evidence-based tool includes items associated with treatment effect estimates in meta-epidemiological studies. The Cochrane Collaboration is developing a similar tool for assessing risk of bias in observational studies, but evidence about the characteristics associated with treatment effect estimates in observational studies is lacking. Our study represents a first step in providing such evidence by assessing the association between design and treatment effect estimates. Other meta-epidemiological studies are needed to assess other important characteristics such as sample size and confounding and the possible associations between these characteristics.

## Supporting Information

S1 Table Search strategy. (DOC)

S2 Table Characteristics of included meta-analyses. (DOC)
