# Supplementary material for: Comparison of Estimates between Cohort and Case–Control Studies in Meta-Analyses of Therapeutic Interventions: A Meta-Epidemiological Study
Source: PLoS One. 2016 May 9;11(5):e0154877. doi: 10.1371/journal.pone.0154877 (PMC4861326; doi:10.1371/journal.pone.0154877)
Supplement: S1 Table — (DOC) [file pone.0154877.s001.doc]

# S1 Table: Search strategy

**Pubmed Search Equation**

| 1. | “observational” [tiab] |
| --- | --- |
| 2. | “cohort” [tiab] |
| 3. | cohort studies [mh] |
| 4. | “epidemiologic” [tiab] |
| 5. | “epidemiological” [tiab] |
| 6. | “systematic review” [tiab] |
| 7. | “systematic reviews” [tiab] |
| 8. | “meta-analysis” [tiab] |
| 9. | “meta-analyses” [tiab] |
| 10. | meta analysis [pt] |
| 11. | “overview” [tiab] |
| 12. | #1 OR #2 OR #3 OR #4 OR #5 |
| 13. | #6 OR #7 OR #8 OR #9 OR #10 OR #11 |
| 14. | 12 AND 13 |

*Date of search: 7 January 2014*

Search limits: Published between 1 January 2013 and 31 December 2013.

Citations retrieved: 3602.
