# Supplementary material for: Comparison of Estimates between Cohort and Case–Control Studies in Meta-Analyses of Therapeutic Interventions: A Meta-Epidemiological Study
Source: PLoS One. 2016 May 9;11(5):e0154877. doi: 10.1371/journal.pone.0154877 (PMC4861326; doi:10.1371/journal.pone.0154877)
Supplement: S2 Table — (DOC) [file pone.0154877.s002.doc]

**S2 Table. Characteristics of included m**eta-analyses

| **First author** | **Condition** | **Intervention** | **Outcome** | **No. cohort/ no. case–control studies** | **No. of participants**  **Total, median (min, max)** | **Combined treatment effect estimate** | **Heterogeneity**  **I^2^ (%)** |
| --- | --- | --- | --- | --- | --- | --- | --- |
| Pharmacological interventions | | | | | | | |
| Ahn | Gastro-intestinal dirsorders | Acid suppressive drugs | Gastric cancer | 11 (2/9) | 62778, 1633 (112, 16739) | 1.42 (1.17, 1.73) | 48 |
| Bang | Cardiology | Statins | Recurrent atrial fibrillation | 7 (6/1) | 156146, 319 (62, 89703) | 0.76 (0.66, 0.87) | 81 |
| Billioud | Inflammatory bowel disease | Pre-operative anti-tumor necrosis factor therapy | Post-operative infectious complications | 18 (15/3) | 3179, 141 (26, 413) | 1.31 (0.89, 1.94) | 39 |
| Brinton | Menopause | Continuous-combined menopausal hormone therapy | Endometrial cancer | 14 (5/9) | 712928, 3543 (317, 465362) | 0.83 (0.68, 1.01) | 78 |
| Deshpande | Cirrhosis | Proton pump inhibitors | Spontaneous bacterial peritonitis | 8 (1/7) | 3752, 135 (84, 2601) | 3.21 (2.24, 4.60) | 59 |
| Dong | Pediatrics | Vitamin D intake during early life | Type I diabetes | 8 (2/6) | 18760, 1028 (202, 10447) | 0.71 (0.51, 0.98) | 75 |
| Gong | Inflammatory bowel disease | Thiopurines | Colorectal neoplasia | 19 (10/9) | 73345, 376 (42, 43969) | 0.65 (0.45, 0.92) | 64 |
| Havrilesky | Contraception | Oral contraceptive pills | Ovarian cancer | 26 (7/19) | 4644724, 2021 (206, 2410072) | 0.74 (0.66, 0.83) | 77 |
| Lee | Osteoporosis | Oral or intravenous bisphosphonates | Osteonecrosis | 12 (7/5) | 1279084, 4479 (111, 441494) | 2.31 (1.37, 3.89) | 91 |
| Phung | Type II diabetes | Sulphonylureas | Cardiovascular composite outcome (myocardial infarction, stroke, cardiovascular-related hospitalization or cardiovascular  death) | 32 (28/4) | 703241, 4077 (238, 275000) | 1.10 (1.04, 1.17) | 69 |
| Richardson | Elderly | Statins | Alzheimer disease | 13 (10/3) | 766823, 2798 (929, 729529) | 0.72 (0.59, 0.89) | 54 |
| Singh A | Diabetes mellitus | Metformin | Colorectal cancer | 9 (6/3) | 689519, 52698 (1320, 252467) | 0.89 (0.80, 0.99) | 68 |
| Singh B | Diabetes mellitus | Metformin | Hepatocellular cancer | 8 (4/4) | 326455, 764 (100, 191223) | 0.50 (0.34, 0.73) | 81 |
| Singh C | Barret’s oesophagus | Proton pump inhibitors | Oesophageal adenocarcinoma and/or high-grade dysplasia | 7 (5/2) | 2941, 350 (77, 812) | 0.28 (0.11, 0.69) | 82 |
| Singh D | Diabetes mellitus | Statins | Hepatocellular cancer | 8 (5/3) | 1310866, 130488 (2332, 480306) | 0.60 (0.49, 0.73) | 65 |
| Sun | Type II diabetes | Insulin therapy | Colorectal neoplasm | 7 (5/2) | 237113, 10067 (200, 81663) | 1.51 (1.14, 1.99) | 54 |
| Tleyjeh |  | Histamine 2 receptor antagonists (H_2_RAs) | Clostridium difficile infection | 17 (4/13) | 190761, 366 (121, 101796) | 1.51 (1.17, 1.96) | 69 |
| Valkhoff |  | Low-dose acetylsalicylic acid | Upper gastrointestinal bleeding | 11 (1/10) | 67903, 2110 (188, 27694) | 3.07 (2.50, 3.77) | 78 |
| Yuhara |  | Nonsteroidal anti-inflammatory drugs | Diverticular bleeding | 6 (1/5) | 50810, 196 (132, 47210) | 2.85 (1.76, 4.60) | 45 |
| Zhu |  | Aspirin | Age-related macular degeneration | 6 (2/4) | 116031, 3430 (146, 104176) | 1.15 (0.92, 1.44) | 79 |
| Wang A | Trauma | Fluid resuscitation | Overall mortality | 8 (5/3) | 13687, 371 (102, 6855) | 1.18 (1.00, 1.40) | 24 |
| Nonpharmacologic Interventions | | | | | | | |
| Hargreave |  | Medically assisted reproduction | Hematological cancer in children | 13 (6/7) | 2461204, 1789 (330. 2424336) | 1.54 (1.26, 1.88) | 0 |
| Wang B | Adrenal masses | Laparoendoscopic single-site adrenalectomy | Surgical conversion | 3 (1/2) | 160, 40 (26, 94) | 3.97 (0.69, 22.86) | 0 |
